# Adie’s tonic pupil after COVID-19: a case report and literature review

**DOI:** 10.22336/rjo.2024.18

**Published:** 2024

**Authors:** Gülay Yalçınkaya Çakır, Işıl Başgil Paşaoğlu, İhsan Çakır, Banu Solmaz

**Affiliations:** * University of Health Sciences Turkey, Beyoglu Eye Training and Research Hospital, Istanbul, Turkey; ** Ophthalmology Department, Medipol University, Faculty of Medicine, Istanbul, Turkey

**Keywords:** Adie’s tonic pupil, anisocoria, COVID-19, pilocarpine

## Abstract

**Objective:** Various neurological complications have been reported after COVID-19. The study aimed to document an unusual case of Adie’s tonic pupil following COVID-19.

**Methods:** The study was a case report.

**Results:** A 28-year-old female had suffered a flu-like disease about 2 months before and the SARSCoV-2 polymerase chain reaction test at that time was positive. Two weeks after infection she noticed an asymmetry between the pupils. The only pathological finding on examination was anisocoria with a larger left pupil in ambient light. Light reflexes were observed in the right eye, while in the left eye, they were absent. Also, there was no near response in the left pupil. A 0.1% pilocarpine test results validated Adie’s pupil diagnosis. After one year of follow-up, the anisocoria decreased but did not completely recover.

**Discussion:** COVID-19 may cause damage to neural structures due to autoimmune ways by activating immune pathways or because of vascular complications that may affect the vasa nervorum. Adie’s tonic pupil is often idiopathic, but it may develop following viral infection.

**Conclusions:** Ocular complications that involve pupil abnormalities may manifest following COVID-19. In the cases of Adie’s tonic pupil, infectious diseases, including COVID-19, should be questioned.

**Abbreviations:** RT PCR = reverse transcription polymerase chain reaction

## Introduction

COVID-19, caused by the SARS-CoV-2 virus, exhibits a broad spectrum of severity and prognostic outcomes. While respiratory symptoms are the most common and notable presentation, various neurological manifestations have also been documented [**1**]. Some reported neurological involvements include cases in which the pupil is affected [**2**-**6**]. An additional case of a unilateral tonic pupil following a COVID-19 infection was presented in this manuscript.

## Case report

A 28-year-old female patient visited the outpatient clinic due to noticing pupil asymmetry over the past two months, with no accompanying symptoms. Her family history revealed no significant features.

She stated that she had a fever, headache, and anosmia about two months earlier and that the SARS-CoV-2 reverse transcription polymerase chain reaction (RT PCR) test performed from the nasal swab at that time was positive. The erythrocyte sedimentation level was 32 mm in the first hour and the d-dimer level was 283 ng/ml. Computed tomography of the chest showed no significant abnormality. As a treatment, eight tablets of 200 mg favipiravir twice a day on the first day and three tablets of 200 mg favipiravir twice daily for the next 4 days were given. She also used 100 mg of acetylsalicylic acid per day for ten days. Repeated SARS-CoV-2 RT PCR test from nasal swab on day 14 was negative. About two weeks after the onset of her complaints, she noticed asymmetry between her eyes. No abnormality was observed in the neurological examination, the brain, and orbital magnetic resonance imaging of the patient referred to the neurology clinic for this asymmetry.

On the Snellen chart, the best corrected visual acuity was 1.0 in both eyes. There was no problem in her color vision, her eye movements were painless and free in all directions. The cornea and lens were normal in both eyes. Intraocular pressures were measured as 13 mmHg and 14 mmHg in the right and left eye using the Goldmann applanation tonometer. Upon fundus examination, both eyes exhibited no retinal abnormalities, optic disc margins were sharp, and the neuroretinal rim was vital. In ambient light, there was anisocoria with a larger left pupil. While direct and indirect light reflexes were positive in the right eye, both reflexes were absent in the left eye. Also, there was no near response in the left pupil.

**Fig. 1:**
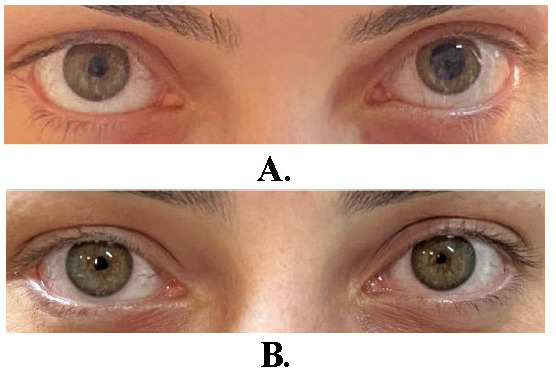
**A.** In ambient light, the right pupil is dilated; **B.** After administration of 0.1% pilocarpine, the left pupil constricts

Based on this anamnesis and examination, a preliminary diagnosis of left Adie’s tonic pupil was contemplated in the patient, and a diluted pilocarpine (0.1%) test was performed to confirm this diagnosis. Half an hour after the instillation of 0.1% pilocarpine in both eyes, it was observed that the left pupil became considerably smaller due to denervation hypersensitivity (**Fig. 1**). This change in pupillary size was also quantitatively demonstrated with the pupillometry module of the Sirius (CSO, Italy) device (**Table 1**).

**Table 1:** The pupillary size of the left eye before and after the diluted pilocarpine test, measured with the pupillometry module of the Sirius (CSO, Italy) device

		Scotopic (0.04 lux)	Mesopic (4 lux)	Photopic (40 lux)
First Visit	Before (mm)	6.40	6.19	6.03
	After (mm)	1.68	1.68	1.67
Year 1 Visit	Before (mm)	4.02	4.01	3.97
	After (mm)	2.07	2.02	2.01

Although the asymmetry between the pupils decreased at the first-year follow-up visit, it was still present. **Table 1** displays the pupillary sizes at one year.

## Discussion

Adie’s tonic pupil, also known as Adie’s syndrome, occurs due to parasympathetic postganglionic denervation [**7**]. Most cases involve women in their thirties [**8**]. Unilateral occurrence is noted in approximately 80% of cases [**8**]. In ambient light, the pupil is more dilated on the affected side, with weak or absent light reaction. When focusing near, tonic constriction (light-near dissociation) can be present. The condition is called Holmes-Adie syndrome when accompanied by reduced deep tendon reflexes [**7**]. While not entirely specific, the presence of miosis due to denervation hypersensitivity on the affected side with the diluted pilocarpine test supports the diagnosis [**7**]. Although it is often idiopathic, it may develop following viral infection or trauma. It has been suggested to be associated with migraine in some cases. 

SARS-CoV-2 is a viral agent that invades neural networks and has many complications. Parasympathetic dysfunction and, as a result, delay and slowing of the pupillary response to light have been observed after COVID-19 [**9**,**10**]. COVID-19 may cause damage to neural structures due to autoimmune ways by activating immune pathways or because of vascular complications that may affect the vasa nervorum. So far, 5 cases of developing tonic pupils after COVID-19 have been reported (**Table 2**) [**2**-**6**]. And 3 were unilateral, like our case [**2**-**4**]. Gopal et al. and Tutar et al. reported cases of unilateral tonic pupil with no other pathology after COVID-19 infection [**2**,**3**]. Three weeks following the COVID-19 infection, Ordás et al. noted the presence of unilateral tonic pupil concurrent with trochlear nerve palsy [**4**]. Ortiz-Seller et al. reported a case of bilateral Adie’s pupil and multifocal choroiditis presenting with visual impairment two days after the onset of symptoms of COVID-19 infection [**5**]. Quijano-Nieto et al. documented a case of bilateral tonic pupil with vertigo two weeks after infection with COVID-19 [**6**]. Long-term follow-up of any of the cases reported in these studies was not mentioned.

**Table 2:** Review of pupillary complications associated with COVID-19 infection

Case report	Time from COVID-19 infection to symptom appearance	Involvement	Accompanying sign
Gopal [**2**]	3 weeks	Unilateral	None
Tutar [**3**]	2 months	Unilateral	Absent deep tendon reflexes in the upper and lower limbs
Ordás [**4**]	3 weeks	Unilateral	Trochlear nerve palsy
Ortiz-Seller [**5**]	2 days	Bilateral	Chorioretinitis
Quijano-Nieto [**6**]	17 days	Bilateral	Peripheral vertigo, persistent anosmia and headache

## Conclusion

In this case, the presence of a tonic pupil after COVID-19 might have been incidental. However, given the timing of her symptoms, the established link between tonic pupil and viral diseases, and the reports of COVID-19 neurological connections, we suspected that our patient’s tonic pupil might have been triggered by COVID-19, possibly due to a postinfectious immune response or vasculopathy.

This one-year follow-up case involved an uncommon type of ocular involvement that may have been caused by SARS-CoV-2. Further reports are needed to accurately define the full clinical context of the neurological effects of COVID-19.

**Conflict of Interest Statement**

The authors state no conflict of interest regarding the research, authorship, and/or publication of this article.

**Informed Consent and Human and Animal Rights Statement**

The subject provided prior informed consent. Written informed consent was obtained from the patient to publish this case report and any associated images.

**Authorization for the use of human subjects**

The study adheres to relevant national regulations and institutional policies concerning human research, following the principles of the Helsinki Declaration. Ethical approval was deemed unnecessary. 

**Acknowledgments**

None.

**Sources of Funding**

The authors receive no financial support for conducting the research, writing the manuscript, or publishing this article.

**Disclosures**

None.
